# Evaluation of Risk Factors Affecting Substance Use among Tenth-Grade Students

**DOI:** 10.1155/2018/1407649

**Published:** 2018-03-15

**Authors:** Dilek Öztaş, Aydan Kalyon, Ayşin Ertuğrul, Çetin Gündoğdu, Hüseyin Balcıoğlu, Yasemin Sağlan, Uğur Bilge, Sevilay Karahan

**Affiliations:** ^1^Department of Public Health, Faculty of Medicine, Yıldırım Beyazıt University, Ankara, Turkey; ^2^Ordu Provincial Health Directorate, Communicable Disease and Control Programs Branch, Ordu, Turkey; ^3^Ordu Provincial Health Directorate, Noncommunicable Diseases Program and Cancer Branch, Ordu, Turkey; ^4^Ordu Provincial Health Directorate, 112 Emergency Command and Control Center, Ordu, Turkey; ^5^Department of Family Medicine, Faculty of Medicine, Eskişehir Osmangazi University, Eskişehir, Turkey; ^6^Eskişehir Public Health Directorate, Eskişehir, Turkey; ^7^Department of Biostatistics, Faculty of Medicine, Hacettepe University Faculty of Medicine, Ankara, Turkey

## Abstract

**Aim:**

The aim of this study is to detect the prevalence of substance use among tenth-grade students; their thoughts, attitudes, behaviors, and tendencies towards substance use; and risk factors of substance use in tenth-grade students in general.

**Methods:**

This study is descriptive and cross-sectional conducted between April and May 2016. Research population consists of tenth-grade students in 2015-2016 school year in the city of Ordu. Since the study involved all tenth-grade students, no sampling was done. Questions on substance use were prepared by Ordu Public Health Directorate and the authors by making use of European School Survey Project on Alcohol and Other Drugs (ESPAD) study questions, AMATEM's “Drugs and Addiction Youth Survey” study conducted on May 1996, and scientific studies conducted previously on similar subjects.

**Results:**

9825 tenth-grade students in 88 schools from 19 counties in the city of Ordu were included in the study. 8714 of the students participated in the survey. Being male, being over the age of 15, mother and father being separated, living with relatives, being in low income, negative feelings about school, perception of being unsuccessful in school, failing a year, absenteeism, and not being content with life are the risk factors for substance use.

**Conclusions:**

The tendency of illegal substance use becoming more and more prevalent especially among youth requires the development of new treatment strategies.

## 1. Introduction

Production, trade, and consumption of illegal substances and related problems increase daily throughout the world; and the average age of users has decreased. Substance use and addiction are an important public health problem rapidly spreading around the world that threatens the users, their families, environments, and the society as a whole, causing health problems and devastating societies in psychological and economical terms with an ever more decrease in the age of users. In this context, substance use and addiction as an overall worldwide issue have become an important problem for our country as well [1, 2]. There are two crucial findings concerning the studies on substance use: there has been a rapid increase in in USA and in our rates of substance use among the adolescents (high school students) country in recent years; and there has been a decrease in tobacco and alcohol use, while the age of first substance use has become younger [3, 4].

According to a report published by UNODC (United Nations Office on Drugs and Crime), age of first volatile substance use is approximately 11, whereas average age of first cannabis use is 16, and average age of first ecstasy use is 17 years worldwide [5]. It has been found that the age of first substance use in Turkey is 14 [6]. Adolescents constitute the most important risk group in substance use. The most frequent substance use disorders among adolescents are harmful use and abuse. Addiction is rarely seen until late adolescence [7]. The programs which may assist in the problem of substance use are prevention and early intervention programs. There are two important reasons with regard to these programs: labor spent and cost of substance use prevention programs are less than the labor and cost spent for treatment and reintegration of substance addicts. Prevention of use in early stages provides for the reduction in substance addiction and increase in lifetime [8].

In order for the substance use prevention programs to be successful in adolescence, it is necessary to determine the children and adolescents under risk and to know what kind of personal and environmental factors create risk in terms of substance use in adolescence [4–7].

The aim of this study is to detect the prevalence of substance use among tenth-grade students; their thoughts, attitudes, behaviors, and tendencies towards substance use; and risk factors of substance use in tenth-grade students in general. Furthermore, treatment and rehabilitation of substance addicts by establishing the early periods of substance use, making a contribution to the preventive programs for children and their families under risk, and supplying data to struggle against addiction are among the objectives of the study.

## 2. Materials and Method

This study is descriptive and cross-sectional conducted between April and May 2016.

### 2.1. Research Population and Sample Size

Research population consists of tenth-grade students in 2015-2016 school year in the city of Ordu. Since the study involved all tenth-grade students, no sampling was done. In our study Admission Criteria and Criteria for Exclusion are as follows.

#### 2.1.1. Study Admission Criteria

- To be a tenth-grade registered student in 2015-2016 school year in the city of Ordu
- To have given consent to participate in the study, minors given consent were provided by parents.

#### 2.1.2. Criteria for Exclusion

- Explicit discrepancy and contradiction in survey responses
- More than 50% of survey answers left unanswered.

### 2.2. Data Collection

*Survey.* Questions on substance use were prepared by Ordu Public Health Directorate and the authors by making use of European School Survey Project on Alcohol and Other Drugs (ESPAD) study questions, AMATEM's “Drugs and Addiction Youth Survey” study conducted on May 1996, and scientific studies conducted previously on similar subjects.

There are a total of 35 questions in the survey. Distribution of questions are as follows: Twelve of the questions are about sociodemographic characteristics, two are about the personal moods of the students, four are about school life, two are about cigarette smoking and alcohol use, six are about addiction status of the family and friends, and nine are about illegal substance use.

### 2.3. Data Collection Method

The data were collected by a survey applied on the students who agreed to participate in the study on a voluntary basis. The survey was conducted on May 4, 2016, simultaneously in the city center and each district.

The research was conducted by a coordinator teacher working in the school with training on substance use (in most of the schools, school counselors performed the duty) and teachers working as survey conductors who were preferably trained in the area and selected by the coordinator teacher. In order to increase the reliability of the research procedure, we made a point of not assigning class masters to carry out the procedure in their own classes.

Necessary training was given to teachers prior to the study in each county separately between April 12, 2016, and April 26, 2016, in places determined by the Ordu Public Health Directorate and by the Provincial Directorate for National Education. In accordance with the distribution lists prepared following the training, questionnaire forms prepared separately for each school were delivered to the school coordinator.

Students answered the survey questions in their classrooms under the supervision of the assigned survey conductors. Prior to the distribution of the survey, survey conductors informed the students of implementation of the survey including the annotations and emphasized especially that personal information would be kept confidential.

The surveys were filled anonymously, and questionnaire forms were collected randomly after completion.

Each survey conductor delivered the charts informing about the class size, number of students absent in class, and the number of questionnaire forms collected, to the school coordinator who in turn made an inventory of the data based on the surveys.

The questionnaire forms were put in plastic files or yellow envelopes separately so that the classes would not get confused and, then, collectively put in a big envelope or package (closed and with the school name visibly on top) and delivered to the county Public Health Centers without any delay (in two days).

Surveys collected in District Community Health Centers were submitted to Ordu Public Health Directorate without any delay with a written report. We have also obtained administrative permits and ethical approval for the use of data for publication purposes.

### 2.4. Evaluation of the Data and Statistical Analysis

The data collected for the study were evaluated via SPSS 18.0 (Statistical Package for Social Sciences) package program. Descriptive findings were expressed in numbers and percentage distributions.

Regarding the analysis of the explanatory findings and the comparison of percentages in independent groups, significance test (chi square test) of the difference between two independent ratios was used and the significance level was accepted as *a* = 0.05.

Additionally, binary logistic regression (Forward: Conditional) analysis was carried out by using variables that were found to have a significant correlation (*p* < 0.05) with substance use in the chi square test (23 variables).

In logistic regression, dependent variable was measured on a dichotomous scale as user and nonuser of any substance in their lifetime, whereas independent variables were measured in dichotomous or nominal terms.

## 3. Results

### 3.1. Descriptive Results

9825 tenth-grade students in 88 schools from 19 counties in the city of Ordu were included in the study. 8714 of the students participated in the survey. The rate of participation was 88,7%. Surveys of 8714 students out of 8399 were deemed valid. The distribution of schools and students participated in the study in the city according to school characteristics is given in Table 1.

48,5% of participants in the study were males and 51,5% were females. Most of the participants were in 16 years' age group (70,4%) whereas 17,4% were in 15, and 11,9% were in 17 years' age group. 0,3% of the students were in 14 years' age group, and the least number of the participants was in this group (Table 2).

While the ratio of students whose mothers were not alive was 1,2%, ratio of students whose fathers were not alive was 3,3%. The ratio of participants whose mothers and fathers were separated was 8,1%. While 83,0% of the participants live with their family, 1,5% live with their relatives. The ratio of the participants living in state or private dormitories was 15,2%. While 17,1% of the students perceived the income level of their family as low, 14,5% perceived it as high. Majority of the students (67,8%) stated that the income level of the family was equal to the expenses. While the ratio of the participants living with five or more people was 51,6%, the ratio of participants living with less than five people was 48,4%. The education level of the mother and father was mostly primary school or lower (58,3% and 38,3%, resp.). 80,7% of the mothers were unemployed. Employment ratio of the fathers was 83,3% (Table 3).

Ratio of the participants who have positive feelings about their school was 43,2% whereas the ratio of the students with negative feelings was 55,5%. 0,7% of the students did not give any opinion or an answer to this question. 8,8% of the participants stated that they failed a year and 33,2% stated that they were absent in the past month. Moreover, only 39,4% thought they were successful in school.

Ratio of participants who were content with life was 60,7%. The ratio of participants who gave a negative answer to this question was 23,4%, and 15,9% of the participants did not give their opinion. The ratio of cigarette smoking in the family was 62,1% whereas ratio of alcohol use in the family was 12,1%. The ratio of cigarette smoking among friends was 70,4%; the ratio of alcohol use was 32,9%. Substance use in the family was 2,6% whereas the ratio was 10,2% for friends.

12,2% of the participants stated they smoke. The ratio of participants who stated they tried smoking was 27,1%, whereas 60,7% never smoked.

Similarly, 5,5% stated they use alcohol, and 13,8% stated they have tried alcohol once, whereas 81,1% never tried alcohol.

Ratio of participants who agreed with the statement “My willpower is strong I wouldn't be addicted even if I used” was 22,4%.

Ratio of participants who agreed with the statement “There is no harm in using drugs once” was 6,2%.

Ratio of participants who agreed with the statement “If people want, they can control narcotic substance use” was 38,0% (Table 4).

While 97,5% of the participants answered no to the question of whether there was anyone offering, selling, or giving drugs within the school premises in the city of Ordu; 2,2% answered yes and 0,3% did not answer.

293 out of 8399 tenth-grade students stated that they have used a substance at least once in their lifetime. The ratio of any substance use at least once in lifetime among tenth-grade students was 3,5%. 234 out of 293 participants who stated they have used a substance at least once in their lifetime answered the question regarding the “number of use in the last 30 days.” Approximately half of the users (124 people) stated that they used substance 1-2 times within the past month. 261 out of 293 participants responded to the question regarding the supplier of the substance. 59,4% of the users (174 people) stated that they acquired it from their friends. Trying (curiosity) occupied the first place (91 people) as a reason for substance use (Table 5).

Evaluation of substance use according to the school characteristics demonstrated that vocational and technical Anatolian high schools had the highest ratio with 4,8% (Table 6).

#### 3.1.1. Correlation between School Characteristics and Substance Use

When substance use according to school characteristics is evaluated, the highest ratio was vocational and technical Anatolian high schools with 4,8%. This relationship between the school characteristics and substance use is also statistically (*p* < 0.001) significant (Table 7).

*In Conclusion.* Studying in vocational technical and Anatolian high schools is a risk factor in terms of substance use.

#### 3.1.2. Gender, Age, and Substance Use Correlation

The rate of substance use was 4,8% in male students and 2,2% in female students. The rate of substance use was higher in males, and the difference between male and female students is statistically significant (*p* < 0.001). While substance use in students of 15 and older (age 16 and 17) was 3,9%, it was 1,8% for students aged 15 and younger (age 14 and 15). Substance use was two times more in students aged 15 and older (age 16 and 17) compared to students aged 15 and younger (age 14 and 15); and the difference is also statistically significant (*p* < 0.001).* In conclusion,* being male and over the age of 15 is a risk factor for substance use.

Substance use in students whose parents are separated (5,3%) was significantly (*p* < 0.01) higher compared to substance users whose parents are not separated (%3,3). The rate of substance use in children who perceived their income level as low (5,0%) was significantly higher (*p* < 0.01) than the rate of substance use of the children (or children who stated they tried a substance) who perceived their income level as normal or good. Substance use in children living with relatives (8,2%) was higher than children living with family (3,3%) and living in private or state dormitories (3,6%). On the other hand, there was no significant correlation between substance use and whether their mother or father is alive, number of people they live with, education, and employment status of the parents.* In conclusion,* mother and father being separated, living with relatives, being in low income group are risk factors for substance use.

Substance use in students with negative feelings about their school (4,8%) was much higher compared to others (1,8%) and the difference is statistically significant (*p* < 0.001).

The rate of substance use in students who think they are not successful in school was (6,0%) two times more than students who think they are successful (3,0%) and the difference is statistically significant (*p* < 0.001).

While the substance use in students who failed a year was 7,4% it was 3,1% in students who did not fail a year. Substance use ratio was two times more in students who failed a year and the difference is statistically significant (*p* < 0.001).

Substance use in students with school absenteeism was 6,6%, whereas it was 1,9% in students with good attendance and the difference is statistically significant (*p* < 0.001).* In conclusion,* negative feelings about school, perception of being unsuccessful in school, failing a year, and absenteeism are risk factors for substance use.

Substance use ratio in students who stated they are not content with their life (7,7%) was much higher compared to others (2,2%) and the difference is statistically significant (*p* < 0.001).* In conclusion,* not being content with life is a risk factor for substance use.

#### 3.1.3. Correlation between Substance Use, Smoking, and Alcohol Use in Family and among Friends

When the correlation between tobacco, alcohol, and substance use in family and friends of the children who have used any substance in their lifetime is examined, substance use in students whose family and friends use any substance, tobacco, and alcohol is significantly higher (*p* < 0.001) (Table 8).

*In Conclusion.* Tobacco use in family and among friends, alcohol use in family and among friends, substance use in family and among friends are risk factors for substance use.

Substance use in children who smoke was 15,7%, while it was 0,6% in students who do not smoke. Substance use in children who use alcohol was 25,5% while it was 1,3% in students who do not use alcohol. The difference is statistically significant (*p* < 0.001).

*In Conclusion.* Tobacco and alcohol use are risk factors for substance use.

Substance use in students who did not agree with the statement “My willpower is strong so I wouldn't be addicted even if I used” was 2,3%, whereas it was 5,2% in students who agreed to the statement. Substance use in students who did not agree with the statement “There is no harm in using drugs once” was 2,5%, whereas it was 11,6% in students who agreed with the statement. Substance use in students who did not agree with the statement “If people want, they can control narcotic substance use” was 2,4%, whereas it was 4,4% in students who agreed with the statement. For all three statements, substance use was significantly higher in students who agreed with the statements compared to those who did not. The correlation is especially strong for the second statement.* In conclusion,* having wrong beliefs and thoughts regarding substance use and addiction is a risk factor for substance use.

In the first stage, regarding the analysis of the data collected, the correlation between the variables was analyzed using the* “chi square test.”* According to the analysis, 23 variables were detected to have a statistically significant correlation with substance use. The variables (risk factors) that increased the substance use significantly are as follows:

- Studying in vocational technical and Anatolian high school
- Studying in the center of Altınordu
- Being male
- Being over the age of 15
- Mother and father being separated
- Living with relatives
- Being in the low income group
- Having negative feelings about school
- Believing they are not successful in school
- Failing a year
- Absenteeism
- Not being content with life in general
- Tobacco use in family
- Tobacco use among friends
- Alcohol use in family
- Alcohol use among friends
- Substance use in family
- Substance use among friends
- Smoking
- Alcohol use
- Having prejudices regarding substance use and addiction and the nature of addiction, agreeing with wrong beliefs and thoughts stated below:
   
*Statement  1. *“My willpower is strong so I wouldn't be addicted even if I used.”
   
*Statement  2.* “There is no harm in using drugs once.”
   
*Statement  3.* “If people want, they can control narcotic substance use.”

In the second stage of the analysis, a stronger and more reliable and advanced statistical analysis technique of logistic regression analysis was conducted for the 23 variables that were found to have a significant correlation with substance use in the chi square test. In the logistic regression analysis, it was found that only 7 out of the 23 variables that have a significant effect on substance use had a significant correlation with substance use in chi square test. The variable county where the* school is located* was classified in four groups as Altınordu center, Fatsa, Ünye, and other peripheral counties. When the odds ratios (OR) of the variables were examined, it was found that compared to Altınordu center odds ratio in other peripheral counties was 0,599, odds ratio in Fatsa was 0,664, and it was 01,021 in Ünye. Odds ratio and the confidence intervals are given in Table 9.

According to the findings, the* risk factors* that affect substance use of tenth-grade students are as follows:

1. Not being content with life
2. Substance use in the family
3. Substance use among friends
4. Tobacco use
5. Alcohol use
6. Agreement with the statement “There is no harm in using drugs once”
7. School being in Altınordu center (Table 9).

## 4. Discussion

Recently, the problem of substance use and abuse has come to the fore in Turkey. When epidemiological and other records are examined, it is reported that the rate of substance use in Turkey is lower than the rate of substance use in European countries and United States of America; however, there has been an increase in the prevalence of substance use [7–10]. There are epidemiologic studies for evaluating the prevalence of substance use in our country, even though they are small in number [7–12].

Among the tenth-grade students in the city of Ordu, the ratio of* any substance use at least once in life* was found to be 3,5%. 12,2% of the participants stated they smoke while 5,5% stated they use alcohol. Adolescence period is characterized by pubertal maturation, continuation of brain development, changes in social roles, and increase in risky behavior and substance use is frequent. In Turkey, the ratio of trying an illegal substance at least once in life among 10th-grade students was 10% [13]. Substance use in adolescence is important because it disrupts cognitive, physical, and psychological development and increases the risk of addiction and medical problems [14, 15].

Often a psychiatric disease accompanies substance use in adolescents. In a public sample study, it was shown that at least one psychiatric disorder accompanied substance addiction in 90% of adolescents under the age of 15; and the most frequent accompanying psychiatric disorders were conduct disorder (72.4%), attention deficit disorder with hyperactivity (63.6%), and depression (52.7%) [4].

A national research conducted in the USA with approximately 70.000 participants at the age of 12 and older [7] demonstrated that the rate of illegal substance use within the last month was 10.1% in the year 2010, 10.1% in the year 2011, 9.5% in the year 2012, and 8.8% in the year 2013. The most commonly used substances among the American youth were alcohol, tobacco, and cannabis [16].

Participants who stated they have used a substance at least once in their life have stated that

- they tried/used the substance out of curiosity and to get rid of their problems,
- they acquired the substance most frequently from friends.

In the logistic regression analysis, it was found that* only seven variables* had a significant effect on the substance use.

Accordingly, risk factors that are effective in substance use in tenth-grade students in the city of Ordu were as follows:

1. Not being content with life
2. Substance use in family
3. Substance use among friends
4. Tobacco use
5. Alcohol use
6. Agreement with the statement “There is no harm in using drugs once”
7. School being in Altınordu center.

The odds ratio of the variable “being content with life” was found to be 1,724 (the variable was classified as Yes/No Opinion/No). According to this, the probability/risk of substance use was 1,724 times more in students who are not content with life compared to students who are content with life.

The odds ratio of the variable substance use in the family was found to be 3,055 (the variable was classified as “Yes, there is” and “No, there is not”). According to this, probability/risk of substance use was 3,055 times more in children with substance use in the family compared to ones without. The odds ratio of the variable substance use among friends was found to be 3,092 (the variable was classified as “Yes, there is” and “No, there is not”).

According to this, probability/risk of substance use was 3,092 times more in children with substance use among friends compared to children without substance use among friends.

The odds ratio of the variable tobacco use was found to be 4,586 in students who tried once and 7,118 in students who are smokers (the variable was classified as No, never smoked; No, but tried once; Yes, I smoke). Probability/risk of substance use in children who agreed with the statement “If people want, they can control substance use” was 2,055 times more compared to those who do not agree with the statement (the variable was classified as “I do not agree,” “I agree”)

Parents' alcohol or substance use, parents neglecting the child, domestic violence, broken family, inability of parents with multiple children to tend to their children's problems enough, conflict in child care approaches, extreme discipline or extreme lack of discipline, and not being interested enough in the education and future of the child increase the risk of substance use [17]. In groups with substance use, parental control was found to be lower compared to groups without substance use [18]. Starting substance use at an early age, childhood trauma, and being neglected [19] increase the risk of substance addiction [20].

Even though substance use among primary and secondary education students in Turkey was found to be lower compared to other countries, it was observed that tobacco use is prevalent. During adolescence, alcohol and tobacco use, insufficient and unbalanced nutrition, use of addictive substances, unsafe sexual activity, and violence are risky health behaviors [21] and they tend to increase during this period [22]. The effort of adolescents to be accepted among friends, the desire to be treated as an adult, and effort to create a new identity result in risk taking and risky health behaviors [21, 23–27].

In a study done with adolescents, it was determined that there was a significant difference between domestic conflict and problematic behavior, and the finding was interpreted as increasing negative behaviors such as alcohol, tobacco, and narcotic substance use [22]. It was demonstrated that adolescents who make use of their time well, who have a close relationship with their parents, who are successful and committed in school show less risky behaviors whereas being in a state of constant anxiety and despair are significant variables in risky behaviors [22–24]. Events that are experienced and acquired habits and behaviors during adolescence have long term results that affect the life of individual [21].

Substance use which is an important public health issue has negative psychological and biological effects on adolescents and the rate of substance use has increased in our country. Negative peer pressure, media messages, and family problems can cause tobacco, alcohol, and drug use in adolescents. Tobacco, alcohol, and narcotic substance use is an important public health problem [25, 26].

Substance use is an important public problem both in developed and in developing countries, and it causes serious health problems. Cannabis, cocaine, nonmedical use of medicine for sleep disorders (or hypnotics) and benzodiazepines, and stimulants such as amphetamines are among the most frequently used psychoactive substances besides tobacco and alcohol [27]. The rate and form of substance use change depending on gender, society, and country; and, according to 2012 estimates, it was stated that 243 million people (approximately 5.2% of the population) between the ages of 15 and 64 used cannabis, cocaine, opioid substances, or amphetamine [28].

Symptoms of drug use are loss of appetite, sudden changes in mood, problems at school or work, risky behaviors, and problems with coordination, attention, and memory and families should be informed and cautious about these factors [29]. Adolescents begin substance use with substances that are easier to find. Substance use begins with tobacco and alcohol may be followed by narcotic substances in the following stages. Cannabis is the most frequently used among illegal drugs and usually evolves from tobacco and alcohol use, which are followed by cannabis use, which is followed by narcotic use [30, 31].

Limitations of the study can be described as follows. Conducting studies evaluating the frequency of substance use disorders and influencing factors is difficult, since people who use substances tend to hide it; and it is difficult to approach groups using substances. Moreover, since our study was done using surveys, contrary to determining the substance users, it is not easy to detect people who are addicted to substances.

In conclusion, not being content with life, substance use in the family, substance use among friends, tobacco use, alcohol use, and agreement with the statement “There is no harm in using drugs once” are the main risk factors. The tendency of illegal substance use becoming more and more prevalent especially among youth requires the development of new treatment strategies [32, 33]. Priority of protective measures should not be glossed over in planning alternative treatments.

## Figures and Tables

**Table 1:** Distribution of schools and students participated in the study in the city according to school characteristics.

City of Ordu	High school	Science high school	Anatolian high school	Vocational and technical Anatolian high school	Religious vocational high school	Total
Number of schools	2	4	40	25	17	88
School ratio (%)	2.0	5.0	46.0	28.0	19.0	100.0
Number of tenth-grade students	287	481	2990	3033	1608	8399
Ratio of tenth-grade students (%)	3.4	5.7	35.6	36.1	19.1	100.0

**Table 2:** Distribution of participants according to gender and age.

	Number	%
Gender		
Female	4322	51.5
Male	4066	48.5
Age		
Age: 14	27	0.3
Age: 15	1459	17.4
Age: 16	5909	70.4
Age: 17	1000	11.9

Total	8399	100.0

**Table 3:** Distribution of participants according to sociodemographic characteristics.

	Number	%
Is the mother alive?		
Yes	8301	98.8
No	97	1.2
Is the father alive?		
Yes	8121	96.7
No	277	3.3
Are mother and father separated?		
Separated	683	8.1
Not Separated	7712	91.8
Life style		
Lives with family	6969	83.0
Lives with relatives	122	1.5
Lives in state dormitory	1118	13.3
Lives in private dormitory	161	1.9
Number of people living together		
2 people	263	3.1
3 people	945	11.3
4 people	2624	31.2
5 and more	4330	51.6
Mother's education status		
Illiterate	560	6.7
Literate	373	4.4
Primary school	3964	47.2
Middle school	1840	21.9
High school	1205	14.3
College-university	326	3.9
Father's education status		
Illiterate	112	1.3
Literate	206	2.5
Primary school	2900	34.5
Middle school	2071	24.7
High school	1945	23.2
College-university	803	9.6
Mother's employment status		
Yes	1609	19.2
No	6780	80.7
Father's employment status		
Yes	7036	83.8
No	1350	16.1
Income status		
Income exceeds expenses	1218	14.5
Income equals to expenses	5698	67.8
Income is less than expenses	1437	17.1

Total	8399	100.0

**Table 4:** Distribution of belief and thoughts regarding substance use.

	Number	%
Statement 1 “My willpower is strong so I wouldn't be addicted even if I used”		
I don't agree	5030	59.9
I agree	1885	22.4
I don't know	1451	17.3
No answer	33	0.4
Statement 2 “There is no harm in using drugs once”		
I don't agree	7428	88.4
I agree	522	6.2
I don't know	419	5.0
No answer	30	0.4
Statement 3 “If people want, they can control narcotic substance use”		
I don't agree	3990	47.5
I agree	3188	38.0
I don't know	1186	14.1
No answer	35	0.4

Total	8399	100.0

**Table 5:** Substance use status.

	Number	%
Any substance use to date		
Yes	293	3.5
No	8106	96.5
Substance use in the past 30 days		
1-2 times	124	53.0
3–5 times	28	12.0
6–9 times	5	2.1
10–19 times	19	8.1
20–39 times	15	6.4
40 or more times	43	18.4
Answered by	234 people	
Those from whom the substance is acquired		
Brother/sister	30	10.2
Friends	174	59.4
A stranger	57	19.5
Answered by	261 people	
Reasons for substance use		
To try (curiosity)	91	31.1
Fun	31	10.6
Boredom	26	8.9
Because friends are using	16	5.5
No special reason	37	12.6
To get away from problems	50	17.1
To calm down when angry	18	6.1
To sleep comfortably	1	0.03
Family influence	6	2.0
Answered by	276 people	

**Table 6:** Distribution of substance use according to school characteristics.

City of Ordu in general	High school	Science high school	Anatolian high school	Vocational and Anatolian high school	Religious vocational high school	Total
Number of tenth-grade students	287	481	2990	3033	1608	8399
Substance users (number)	10	8	88	145	42	293
Ratio of substance users	3.5%	1.7%	2.9%	4.8%	2.6%	3.5%

**Table 7:** Substance use according to school characteristics.

School characteristics	Any substance to date in life				Total		*x* ^2^/*p*
	User		Nonuser				
	Sayı	%	Sayı	%	Sayı	%	
High school	10	3.0	277	97.0	287	100.0	*x* ^2^: 27.17 *p* < 0.001
Science high school	8	1.7	473	98.3	481	100.0	
Anatolian high school	88	2.9	2902	97.1	2990	100.0	
Vocational technical Anatolian HS	145	4.8	2888	95.2	3033	100.0	
Religious vocational high school	42	2.6	1566	97.4	1608	100.0	
Total	293	3.5	8106	96.5	8399	100.0	

**Table 8:** Distribution of substance use according to substance, tobacco, and alcohol use in family and among friends.

	Any substance used to date in life				Total		*x* ^2^/*p*
	User		Nonuser				
	Number	%	Number	%	Number	%	
Tobacco use in family							
Yes	220	4.2	4994	95.8	5214	100.0	*x*2; 22.49 *p* < 0.001
No	72	2.3	3108	97.7	3180	100.0	
Total	292	3.5	8102	96.5	8394	100.0	
Alcohol use in family							
Yes	102	10.0	914	90.0	1016	100.0	*x*2; 148.15 *p* < 0.001
No	190	2.6	7187	97.4	7377	100.0	
Total	292	3.5	8101	96.5	8393	100.0	
Tobacco use among friends							
Yes	271	4.6	5643	95.4	5914	100.0	*x*2; 72.24 *p* < 0.001
No	21	0.8	2450	99.2	2450	100.0	
Total	292	3.5	8093	96.5	8385	100.0	
Alcohol use among friends							
Yes	221	8.0	2545	92.0	2766	100.0	*x*2; 249.08 *p* < 0.001
No	71	1.3	5541	98.7	5612	100.0	
Total	292	3.5	8086	96.5	8378	100.0	
Substance use in family							
Yes	53	23.9	169	76.1	222	100.0	*x*2; 284.61 *p* < 0.001
No	237	2.9	7925	97.1	8162	100.0	
Total	290	3.5	8094	96.5	8384	100.0	
Substance use among friends							
Yes	155	18.1	703	81.9	858	100.0	*x*2; 603.59 *p* < 0.001
No	137	1.8	7378	98.2	7515	100.0	
Total	292	3.5	8081	96.5	8373	100.0	

**Table 9:** Logistic regression analysis results comprising the variables that affect substance use status of students.

Variables	B	Stan. Err.	Wald	df	*p*	(OR)	95% confidence interval	
							Lowest	Highest
Content with life status reference: Content	.545	.143	14.501	1	<0.001	1.724	1.303	2.282

Substance use in the Family reference: No substance use in the family	1.117	.233	22.891	1	<0.001	3.055	1.934	4.828

Substance use among friends Ref.: No use among friends	1.129	.158	51.227	1	<0.001	3.092	2.270	4.212

Tobacco use reference: No. Never Smoked			61.675	2	<0.001			
Tried once	1.523	.229	44.179	1	<0.001	4.586	2.927	7.186
Yes. I smoke	1.963	.253	59.949	1	<0.001	7.118	4.331	11.698

Alcohol use reference: No. never used			46.164	2	<0.001			
No. but tried once	.712	.182	15.392	1	<0.001	2.039	1.428	2.911
Yes. I use	1.441	.212	46.062	1	<0.001	4.225	2.787	6.405

“If people want, they can control narcotic substance use” Statement reference: Don't agree	.732	.155	21.514	1	<0.001	2.055	1.516	2.787

County where the school is located reference: Altınordu Center			10.047	3	.018			
Other counties	−.512	.216	5.627		.018	.599	.393	.915
Fatsa	−.410	.218	3.533	1	.060	.664	.433	1.018
Ünye	−.021	.171	.015	1	.902	1.021	.731	1.428

Constant	5.612	.226	618.895	1	<0.001	.004		
